# Systemic risk factors contributing to sialolithiasis: a big-data retrospective analysis

**DOI:** 10.1007/s00784-025-06179-7

**Published:** 2025-02-06

**Authors:** Ehud Jonas, Leon Gillman, Daya Masri, Eli Rosenfeld, Gavriel Chaushu, Gal Avishai

**Affiliations:** 1 Department of Oral and Maxillofacial Surgery, The Maurice and Gabriela Goldschleger School of Dental Medicine, The Faculty of Medicine, Tel Aviv University, Tel Aviv, Israel; 2 Department of Oral and Maxillofacial Surgery, Rabin Medical Center, Petah Tiqwa, Israel

**Keywords:** Sialolithiasis, Salivary gland, Big-data, Risk factor, Systemic diseases, Dyslipidemia

## Abstract

**Aims:**

To investigate systemic risk factors contributing to the formation of sialolithiasis.

**Methods:**

A retrospective big-data cohort study was conducted using data from Clalit HMO in Israel. Sialolithiasis patients were identified based on ICD-10 codes and matched with controls. Univariate and multivariable logistic regression analyses were performed to assess the relationship between systemic conditions and sialolithiasis. P-value < 0.05 was considered significant.

**Results:**

5100 sialolithiasis cases were collected. The statistical analysis revealed that hypertension (OR = 1.14, 1.08–1.24, *p* < 0.001), dyslipidemia (OR = 1.33, 1.27–1.41, *p* < 0.001), nephrolithiasis (OR = 1.55, 1.42–1.63, *p* < 0.001) and cholelithiasis (OR = 1.22, 1.16–1.27, *p* < 0.001) were significantly correlated with sialolithiasis.

**Conclusions:**

Systemic diseases, particularly dyslipidemia, play a role in the development of sialolithiasis. Further research is needed to explore the underlying mechanisms linking these conditions and to develop targeted strategies for the prevention of sialolithiasis.

**Clinical relevance:**

This study highlights the potential interplay between systemic conditions and sialolithiasis. Recognizing these associations can inform clinical practice in understanding the pathogenesis of the disease, risk assessment, early diagnosis, and preventive measures.

## Introduction

Sialolithiasis is a condition characterized by deposits of calcified material in the ducts and hilus of the salivary glands. The size of these deposits may vary from 2 mm to more than 1.7 cm which can obstruct the lumen of the duct and interfere with the normal flow of saliva [1–3]. This obstruction may cause sialadenitis which commonly presents with pain, swelling of the salivary gland, purulent discharge, and often systemic symptoms including malaise and fever [2, 3]. Studies that investigated the structure of sialoliths revealed that they are composed from both organic and inorganic materials [4]. The organic component typically includes glycoproteins, cellular debris, and bacteria. The inorganic component primarily consists of calcium phosphates, with varying proportions of different calcium phosphate compounds, including hydroxyapatite, octacalcium phosphate, whitlockite, brushite, and amorphous calcium phosphate [4–6]. These components’ proportions vary depending on the specific gland involved and the distance from the sialolith’s center [6, 7].

The etiology of sialolithiasis is not well understood [8]. In recent years, it has been suggested that the pathophysiology of this condition is caused by increased calcium precipitation due to hyposalivation, anatomical variability, or salivary composition [2, 3]. These etiologies can be further enhanced by risk factors such as submandibular gland [4, 9, 10], tobacco smoking [3, 11–13], geographic location [3, 14–17], dyslipidemia and elevated Body Mass Index (BMI) [3, 13], and alcohol consumption [13].

Although some local and habitual risk factors for sialolithiasis have been studied, the correlation between this condition and systemic diseases has not been widely investigated. Hung et al. (2016) conducted a retrospective study of 745 sialolithiasis patients in Taiwan. They found a correlation between prior diagnosis of sialolithiasis and cholelithiasis, and no correlation with hyperparathyroidism [12]. Wu et al. (2016) investigated the relationship between sialolithiasis and nephrolithiasis in a study of 966 individuals diagnosed with sialolithiasis. They reported that patients with sialolithiasis tended to have higher prevalence of either one or more of the following diagnoses: diabetes, hypertension, renal disease, and nephrolithiasis [17]. Hung et al. (2019) added that patients with osteoporosis and hyperlipidemia had an increased risk of sialolithiasis [18]. However, Jin et al. (2020) reported no statistically significant differences in diabetes, dyslipidemia, and hypertension [13], and Choi et al. (2018) concluded that there was no association between nephrolithiasis and sialolithiasis [19]. Sánchez Barrueco et al. (2022) added that systemic conditions such as nephrolithiasis, cholelithiasis, diabetes mellitus, hypertension and dyslipidemia had no significant correlation with chronic obstructive sialadenitis (COS). While other conditions including radioiodine treatment and autoimmune diseases were significantly correlated to COS [9]. The authors of these articles postulated that the rise in prevalence of sialolithiasis in these conditions may be linked to higher serum concentration of calcium, or to medications inducing hyposalivation [12, 17, 18].

Limited studies with large cohorts have been conducted in an attempt to clarify possible correlations between sialolithiasis and other systemic conditions. The articles reviewed above were conducted in limited geographic locations which may affect their external validity. Therefore, this article aimed to investigate systemic risk factors for sialolithiasis in a large-scale cohort. The null hypothesis was that systemic conditions do not correlate with this condition.

## Methods

A big-data retrospective cohort study was conducted according to the STROBE guidelines (Strengthening the reports of observational studies in epidemiology) [20] from 2005 to 2019. The study received ethical approval from the medical center’s Helsinki committee (RMC-21-0139). Data was extracted from the database of Clalit HMO (Health Maintenance Organization) in Rabin medical center, Petach tikva, Israel and Dan and Petach tikva counties. As of 2020, these databases were comprised of over 1,200,000 individual’s medical records. According to the International Classification of Disease-10 (ICD-10) coding of sialolithiasis (ICD-10 code K11.5), medical records of patients with this condition were collected without human intervention.

Each sialolithiasis case was categorized anonymously and matched by gender and birthdate (± one week) with 3 other control patients who were not diagnosed with the disease using a K- nearest neighbors (KNN) algorithm.

### Inclusion criteria

- Age > 22 years old (This age corresponds to the typical age at which individuals in Israel complete their mandatory military service, during which medical records are generally inaccessible).
- Diagnosis of sialolithiasis according to the ICD-10 code K11.5.
- Registration at Clalit HMO during 2005–2019.

### Exclusion criteria

- Missing demographic and medical data.

### Data collection

- Age.
- Gender.
- Diagnosis of Hypertension.
- Diagnosis of Diabetes Mellitus.
- Diagnosis of Dyslipidemia **(**including Hypercholesterolemia, Hypertriglyceridemia, and Mixed Hyperlipidemia).
- Diagnosis of Cholelithiasis.
- Diagnosis of Nephrolithiasis.

### Statistical analysis

Data were analyzed in R (v4.3) and RStudio (v2023.3). Descriptive statistics summarized the data including means for continuous variables and frequencies for categorical variables. A univariate logistic regression was used to explore the relationship between the presence of one or more of the above listed systemic diseases and sialolithiasis. Variables were included in the multivariable logistic regression following multicollinearity assessment using the variance inflation factor (VIF) analysis and a univariate p-value < 0.1. The threshold for statistical significance was set at *p* < 0.05.

## Results

5,100 sialolithiasis patients and 15,296 matched controls were collected. The mean age of sialolithiasis patients was 55.75 ± 17.16, and 57.78% were females. Regarding the systemic conditions, 35.78% were diagnosed with hypertension, 19.03% had diabetes mellitus, 17.11% had dyslipidemia, 6.19% were previously diagnosed with nephrolithiasis, and 6.99% experienced cholelithiasis. For further details, see Table 1.

**Table 1:** Complete cohort characteristics

Variable	Subgroup	*N*	%	Mean
Total		20,396	100%	
Sialolithiasis		5100	25%	
Gender	Female	11,784	57.78%	
	Male	8,612	42.22%	
Age				55.74 ± 17.16
Hypertension		7297	35.78%	
Dyslipidemia		3491	17.11%	
Diabetes mellitus		3883	19.03%	
Nephrolithiasis		1263	6.19%	
Cholelithiasis		1427	6.99%	

The univariate statistical analysis revealed that hypertension patients had significantly higher odds for sialolithiasis (OR = 1.14, 1.08–1.24, *p* < 0.001), and dyslipidemia increased the odds of sialolithiasis by 22% (OR = 1.22, 1.16–1.27, *p* < 0.001). Patients who had a history of nephrolithiasis had higher odds for having a sialolith by 55% (OR = 1.55, 1.42–1.63, *p* < 0.001), while previous cholelithiasis increased the risk for sialolithiasis by 33% (OR = 1.33, 1.27–1.41, *p* < 0.001). Diabetes mellitus did not significantly increase the risk for sialolithiasis. For further details, see Table 2.

**Table 2:** Univariate statistical analysis

Variable	OR	CI	*P*- value
Hypertension	1.14	1.08–1.24	< 0.001
Dyslipidemia	1.22	1.16–1.27	< 0.001
Diabetes mellitus	-	-	N.S
Nephrolithiasis	1.55	1.42–1.63	< 0.001
Cholelithiasis	1.33	1.27–1.41	< 0.001

*Note*: Gender and Age were not analyzed due to the matching process

After accounting for confounding factors, the multivariable logistic regression was able to predict 5% of the model’s variance (Nagelkerke = 0.05). The Analysis revealed a significant relationship between dyslipidemia and sialolithiasis. Individuals with dyslipidemia had a 35.4% increased risk of for the disease than those with normal serum lipid levels (OR = 1.354, 1.09–1.68, *p* < 0.01).

## Discussion

The relationship between various systemic medical conditions and sialolithiasis is a relatively unplowed field of research. Improved understanding of the role of systemic mechanisms in the pathogenesis of sialolithiasis may aid in developing preventive treatments. This study’s robustness of big-data cohort can shed important light on the contributing factors to the development of sialolithiasis.

The pathophysiology of sialolith formation is not completely understood. It has been proposed that calcium and phosphate ions precipitate within the ducts and hilus of the salivary glands, similar to cholelithiasis and nephrolithiasis formation [2, 3]. Together with altered salivary flow, this process may hasten the formation of a sialolith large enough to obstruct the duct lumen and initiate symptoms of sialadenitis. Elevated calcium ion concentration is a suggested contributing factor in sialolith formation. Since there is a correlation between salivary and serum calcium levels [21, 22], it may be hypothesized that systemic conditions causing elevated serum calcium levels may lead to salivary supersaturation, increasing the likelihood of sialolithiasis in these patients.

Traditionally, sialolithiasis was considered more prevalent in men [9, 23, 24]. This was supported by several studies reporting men-to-female ratio of up to 2:1 [24]. Recent studies have challenged this view with some studies reporting similar or higher rates in females [13, 14, 25, 26]. In the current study, females comprised of 57.4% of cases.

The univariate statistical analysis revealed that hypertensive patients were 14% more likely to have sialolithiasis than normotensive individuals. This result is consistent with previous studies [17, 18]. Hypertension is a condition defined by a systolic and diastolic blood pressures higher than 130 mm/Hg and 80 mm/Hg respectively [27]. It is a major risk factor for ischemic heart disease, stroke, renal failure, and sexual dysfuncion [27]. Hypertension is usually treated using a wide array of medications including beta blockers, angiotensin-converting enzyme inhibitors, and diuretics. These drug classes can induce hyposalivation and altered salivary composition through diverse mechanisms, such as interactions with alpha-2 adrenergic receptors in the salivary glands, alterations in calcium homeostasis, and changes in fluid balance secondary to renal effects [28]. These may promote the formation of sialolithiasis by increasing the concentration of calcium and other minerals in the saliva, and reducing salivary flow [28, 29]. Nevertheless, further studies are needed to investigate the possible correlation between antihypertensive medications and sialolithiasis.

Dyslipidemia is another condition significantly correlated with sialolithiasis. The multivariable statistical analysis showed that individuals with dyslipidemia had significantly higher odds of having a sialolith (OR = 1.354). Previous studies have explored the connection between sialolithiasis and metabolic disorders [3, 13]. These studies have previously reported a potential link to higher BMI and impaired serum lipid levels [3], while others found no significant difference in incidence rates based on BMI [13]. It has been suggested that abnormal lipid levels induce impaired salivary flow and potential hyposalivation [30, 31]. This may contribute to salivary stasis and supersaturation, creating an environment conductive to calcium salt precipitation and sialolith formation.

Nephrolithiasis is a common condition that is characterized by the formation of calcified material in the kidneys, which affects nearly 11% of American men [32]. These deposits may obstruct the ureter or renal pelvis and result in symptoms including pain, hematuria, nausea, urinary urgency, restlessness, and nausea [32]. In the current study, nephrolithiasis significantly increased the odds of having sialolithiasis by 55%. Previous studies have also investigated the link between this condition and sialolithiasis. Wu et al. (2016) conducted a study among 966 sialolithiasis patients. They found that individuals with a prior diagnosis of nephrolithiasis were 4.74 times more likely to develop sialolithiasis [17]. Others reported similar results [33]. Choi et al. (2018) investigated the relationship between nephrolithiasis and other diseases. They collected 24,038 cases of patients with nephrolithiasis and 96,152 matched controls. Their complete cohort had 111 patients diagnosed with sialolithiasis. The statistical analysis did not result in a significant correlation between nephrolithiasis and sialolithiasis [19]. Hemminki et al. (2018) performed a study to assess the familial correlation between stone diseases like nephrolithiasis, sialolithiasis, and cholelithiasis. They concluded that while individuals within the same family had an increased risk for having the same disease, they had lower risk for other types of stone disease doubting the reported comorbidities between the three diseases [34].

Choleliths or gallstones are another type of stone disease characterized by calcified deposits in the gallbladder. There are three types of gallstones: cholesterol, pigmented, and mixed depending on their composition. Pigmented stones comprise about 15% of cholelithiasis cases, and are composed of calcium salts including calcium carbonate, calcium phosphate and calcium palmitate [35]. We found that patients with cholelithiasis had significantly higher chances of having sialolithiasis (OR = 1.33). The relationship between gallstones and sialolithiasis has been investigated in several studies. Hung et al. (2016) conducted a population-based study among 745 incidents of sialolithiasis. They concluded that there is an association between cholelithiasis and sialolithiasis [12]. Kim et al. (2019) studied the relationship between the two stone diseases in a nationwide sample cohort in Korea. Their study revealed an insignificant relationship between the two diseases [26]. In an attempt to assess the impact of cholelithiasis on salivary stone formation, Mortazavi et al. (2024) conducted a meta-analysis of the Kim et al. (2019) and Hung et al. (2016) cohorts. They concluded that cholelithiasis is a significant risk factor for sialolithiasis [35].

Nephrolithiasis, cholelithiasis, and sialolithiasis share several risk factors including hypertension, dyslipidemia, gout, dehydration, and possibly some medications [2, 3, 17, 36, 37]. Some authors highlighted the shared composition of the stone diseases [19, 35]. Therefore, we assume that sialolithiasis, much like other stone diseases, could be triggered by an imbalance in the mineral composition of saliva, specifically a disruption in calcium levels that occurs in nephrolithiasis as well. Further studies are needed to unveil the interplay of mineral metabolism and sialolith formation.

This study has several limitations arising from its retrospective big-data nature. Sialolithiasis is a condition that is usually diagnosed once the patient is symptomatic. Thus, it is likely that a portion of individuals with this condition are not diagnosed, which may underestimate the reported incidence of the disease. Since the data was collected without human intervention, some possible confounding factors including sialolith size and location, familial history, method of treatment and environmental factors could not be collected. Furthermore, the temporal relationship between underlying disease diagnosis and sialolith appearance remains unexplored and beyond this study’s scope. While this study identified a link between these conditions, further research is needed to establish a causal relationship and investigate the timeline of disease progression and sialolith formation.

## Conclusions

This comprehensive study underscores the relationship between several systemic health conditions and sialolithiasis. We can conclude that hypertension, dyslipidemia, nephrolithiasis and cholelithiasis are significant risk factors for sialolithiasis. These findings highlight the need for future studies that will investigate the complex relationship between sialolithiasis and other systemic conditions which may unveil further aspects of the pathophysiology of sialolith formation.

## Data Availability

No datasets were generated or analysed during the current study.
